# Drug repositioning for enzyme modulator based on human metabolite-likeness

**DOI:** 10.1186/s12859-017-1637-5

**Published:** 2017-05-31

**Authors:** Yoon Hyeok Lee, Hojae Choi, Seongyong Park, Boah Lee, Gwan-Su Yi

**Affiliations:** Department of Bio and Brain Engineering, Korea Advanced Institute of Science and Technology (KAIST), 291 Daehak-ro, Yuseong-gu, Daejeon, 34141 South Korea

**Keywords:** Metabolite-likeness, Drug repositioning, Enzyme modulator, Structure similarity, Antimetabolites

## Abstract

**Background:**

Recently, the metabolite-likeness of the drug space has emerged and has opened a new possibility for exploring human metabolite-like candidates in drug discovery. However, the applicability of metabolite-likeness in drug discovery has been largely unexplored. Moreover, there are no reports on its applications for the repositioning of drugs to possible enzyme modulators, although enzyme-drug relations could be directly inferred from the similarity relationships between enzyme’s metabolites and drugs.

**Methods:**

We constructed a drug-metabolite structural similarity matrix, which contains 1,861 FDA-approved drugs and 1,110 human intermediary metabolites scored with the Tanimoto similarity. To verify the metabolite-likeness measure for drug repositioning, we analyzed 17 known antimetabolite drugs that resemble the innate metabolites of their eleven target enzymes as the gold standard positives. Highly scored drugs were selected as possible modulators of enzymes for their corresponding metabolites. Then, we assessed the performance of metabolite-likeness with a receiver operating characteristic analysis and compared it with other drug-target prediction methods. We set the similarity threshold for drug repositioning candidates of new enzyme modulators based on maximization of the Youden’s index. We also carried out literature surveys for supporting the drug repositioning results based on the metabolite-likeness.

**Results:**

In this paper, we applied metabolite-likeness to repurpose FDA-approved drugs to disease-associated enzyme modulators that resemble human innate metabolites. All antimetabolite drugs were mapped with their known 11 target enzymes with statistically significant similarity values to the corresponding metabolites. The comparison with other drug-target prediction methods showed the higher performance of metabolite-likeness for predicting enzyme modulators. After that, the drugs scored higher than similarity score of 0.654 were selected as possible modulators of enzymes for their corresponding metabolites. In addition, we showed that drug repositioning results of 10 enzymes were concordant with the literature evidence.

**Conclusions:**

This study introduced a method to predict the repositioning of known drugs to possible modulators of disease associated enzymes using human metabolite-likeness. We demonstrated that this approach works correctly with known antimetabolite drugs and showed that the proposed method has better performance compared to other drug target prediction methods in terms of enzyme modulators prediction. This study as a proof-of-concept showed how to apply metabolite-likeness to drug repositioning as well as potential in further expansion as we acquire more disease associated metabolite-target protein relations.

**Electronic supplementary material:**

The online version of this article (doi:10.1186/s12859-017-1637-5) contains supplementary material, which is available to authorized users.

## Background

Over the past few decades, due to the high rate of failure in recent drug development processes [1], drug repositioning has emerged as a new paradigm in which a new indication of a drug that is already on the market or of one that failed to be commercialized in the clinical stages is demonstrated [2]. To date, various computational methods have been developed for drug repositioning classified as target-based [3–5], knowledge-based [6–8], signature-based [9–11] and network-based [12, 13] approaches. While these methods have contributed much to drug discovery, the space of innate human metabolite has relatively not been considered in those approaches. The human metabolite space might be a good resource of drug discovery because the structure of a drug could be similar to innate ligands if the drug interacts with the same target or several targets in the same manner as their endogenous counterparts. An example is the human opioid system. Morphine mimics endogenous opioid endorphins, and their pharmacological and physiological effects have been proven to be similar [14]. Another example is the well-known drug aspirin. Aspirin inhibits cyclooxygenase [15], and the drug may be an innate metabolite of humans according to a recent report [16]. Likewise, although the metabolite resemblance of a drug is one of the important features for drug discovery, the search for possible metabolite-like drugs is limited and biased currently.

The ‘metabolite-likeness’ concept was proposed to offer a quantitative evaluation of metabolite-like chemicals as a new druggability filter in that a metabolite-like drug is likely to hitchhike the transporters of endogenous metabolites [17–19]. On the other hand, almost all endogenous metabolites also have interaction partners in terms of metabolic enzymes. Therefore, the metabolite-likeness of a drug would be a good characteristic to predict new enzyme-drug relationships. However, there is no systematic approach for applying metabolite-likeness to predict the drug candidates for enzyme modulators.

In this paper, we applied the ‘metabolite-likeness’ concept to predict enzyme modulators in an existing drug list which may have similar effects as endogenous ligands or metabolites. To this end, we generated a drug-metabolite similarity matrix and checked the global similarity patterns of the metabolite-likeness of the drugs. To validate the metabolite-likeness as a new target prediction method, we carried out a performance test of the metabolite-likeness on a drug-target prediction of the antimetabolite class. In this step, we assumed that the list of antimetabolites is a gold standard positive set because they all resemble innate metabolites by definition. Then, we compared the performance of our method to known drug-target prediction methods including SwissTargetPrediction [20], TargetNet [21], and Libdock algorithm of molecular docking [22]. After showing that our method outperforms the other drug-target prediction methods in terms of drug-enzyme relations, we set the similarity threshold and proposed promising drug candidates for the target enzymes of 10 antimetabolites. In addition, we showed that drug repositioning candidates from our method were supported well by literature evidence. As a result of our research, we demonstrated that metabolite-likeness can be used for new drug-target prediction in the case of enzyme modulator prediction.

## Methods

### Dataset collection

As a human metabolite set, we used intermediary metabolites, which are related to reactions within the cell [23]. We adopted the list of intermediary metabolites from the paper of Steve O′Hagan et al. (See details in [19]). Based on the list, we collected the SDF files of intermediary metabolites from HMDB (Version 3.6) [24], ChEBI [25], and PubChem [26]. The final list of metabolites consisted of 1,110 metabolites.

The list of FDA-approved small molecule drugs was downloaded from DrugBank 5.0 [27] (http://www.drugbank.ca/releases/latest) in July 2016 as an SDF file. The number of approved drugs was increased to 1,861 compared with 1,381 drugs in a previous paper [19].

### Drug – metabolite similarity matrix

To compare the structural distances between drugs and human metabolites, we constructed drug-metabolite similarity matrix. We used the Python 3.5 programming language (Python Software Foundation, http://www.python.org/) with the RDKit module, an open-source cheminformatics toolkit (www.rdkit.org/) [28]. We converted 2D structures to a molecular descriptor; Public MDL MACCS keys fingerprints [29]. It consists of 1,024 bits based on a predefined set of 166 substructures. After converting the SDF files to the fingerprints, the similarity between drug-metabolite pairs was calculated by the Tanimoto similarity (T_c_) which is widely used and easy to calculate. The Tanimoto similarity is generally calculated with the bits of the binary fingerprint vectors:

$$ {T}_c\left( A, B\right)=\frac{C}{A+ B- C} $$

, where A and B are the number of bits present in compounds A and B, respectively, and C is the number of bits shared by A and B [30, 31].

To see global similarity patterns between human metabolites and FDA-approved drugs, we plotted a heat map through hierarchical clustering. For this purpose, we used the gplots library [32] in the R programming language [33]. Hierarchical clustering of row and column was carried out using the complete linkage algorithm. For readability, ten discrete colors were chosen from http://www.colorbrewer2.org/ [34]. Moreover, we highlighted and investigated the clusters that had the following criteria: i) more than 50 drugs, ii) more than 100 metabolites, and iii) almost 30% of the total relations in the cluster had similarity scores of 0.7 or higher.

### Selection of gold standard positive set

An antimetabolite list was obtained from DrugBank’s ‘Antimetabolites’ Medical Subject Headings (MeSH) category and ‘Antimetabolites, Antineoplastic’ MeSH category. Within the list, we considered approved drugs whose targets are human enzymes only, to make the drug–target enzyme–substrate relationships clear. To obtain a filtered drug list, each drug in the antimetabolite list was mapped to their drug targets with the UniProt accession number [35]. After that, the antimetabolites, which have human enzymes as their targets, were filtered with BRENDA [36] in which mapping the UniProt accession numbers to their E.C numbers is available. Finally, using the E.C numbers, we extracted the substrates of each target enzyme from the reaction information of Recon2 [37] and the KEGG human pathway [38]. When we extracted the substrate information, the commonly involved substrates in many reactions, such as water, cofactors, etc. were excluded from selection.

### Performance comparison with other drug-target prediction methods

To assess the performance of metabolite-likeness, we used three different known Drug-Target Interaction (DTI) prediction methods: SwissTargetPrediction (STP) [20], TargetNet (TN) [21], and Libdock algorithm of molecular docking [22].

The SwissTargetPrediction (STP) tool is a well-known target prediction method developed by the Swiss Institute of Bioinformatics [20]. The STP tool compares a query molecule to a compound library of 280,000 molecules active on more than 2,000 targets using a combination of 2D and 3D similarity measures. The STP provides only 15 predicted targets for a query molecule with probability scores as a prediction result. We extracted the SMILES information from the SDF files of 1,861 FDA-approved drugs and submitted them as inputs. The prediction results of the STP were rearranged in a table with a descending order of probability scores for the performance evaluation.

The TargetNet (TN) tool is a recently published drug-target prediction method developed by the Computational Biology and Drug Design Group of Central South University [21]. The TN tool provides a prediction for the activity of a submitted molecule across 623 human proteins on the website by establishing SAR models for DTI profiling and training the models with the biological activity data from Binding DB. We extracted the SMILES information from the SDF files of 1,861 FDA-approved drugs and submitted them as inputs. Among the 7 fingerprint models of TN, we used the MACCS fingerprints to obtain the DTI prediction result. The prediction results of TN, which are the probability scores of the predicted human proteins for the submitted drugs, were rearranged in a table with a descending order of probability scores for the performance evaluation.

The Libdock algorithm [22] in Discovery Studio 3.1 (DS) from Accelrys (San Diego, CA, USA) was used to perform molecular docking. Docking experiments on FDA-approved drugs containing hydrogen atoms were carried out against two proteins, Dihydrofolate reductase (DHFR) and Thymidylate synthase (TYMS), respectively. The X-ray crystal structure complex of DHFR with folate, which was obtained at 2.3 Å, was downloaded from the protein data bank (PDB ID: 1DHF) [39]. Moreover, the X-ray crystal structure complex of TYMS with dUMP and Raltitrexed, one of the active inhibitors, determined at a resolution of 1.9 Å was downloaded from the PDB (PDB ID: 1HVY) [40]. Protein preparation and minimization were carried out in DS. Hydrogen atoms were added to the protein-ligand complex under the CHARm force field. All water molecules were removed and the pH environment was adjusted to neutral. The active sites of each protein were defined with a 10 Å radius around the bound ligands (innate metabolite or modulator). The libdock scores were obtained by the libdock algorithm with the default setting except for calculating the ligand conformations for each drug within an energy range of 10 kcal mol^−1^ above the global energy minimum. In addition, we considered only the maximum libdock scores among several libdock scores in one drug.

Using the ordered drug-target enzyme prediction score lists from each DTI prediction method, we plotted the receiver operating characteristics (ROC) curve of the binary classifier based on the prediction scores from each method. To draw the ROC curve, we used an ROCR library [41] in the R programming language [33].

### Similarity threshold determination for enzyme modulator predictions

To find the optimal threshold for the prediction of enzyme modulators, we calculated similarity scores for all drug-metabolite relations in the finalized list of antimetabolites. Then, the similarity scores of the drug–target enzyme relationships obtained from the drug-metabolite relations, including the gold standard positive relationships, were arranged in a table in descending order of score. Using this ordered drug-target enzyme relation list, we plotted the ROC curve of the binary classifier based on the similarity scores. To draw the ROC curve, we used an ROCR library [41] in the R programming language [33]. We calculated the optimal threshold at which Youden’s J statistics [42] is maximized giving equal weighting for sensitivity and specificity in the ROC curve.

The formula of Youden’s index, J(x), is as follows:

$$ J(x)= S p(x)+ S e(x)-1, $$

where Sp(x) indicates the specificity, and Se(x) denotes the sensitivity of the classifier when a threshold is assigned to a value x.

## Results and Discussions

### Metabolite-likeness of the FDA-approved drugs

**Fig. 1:**
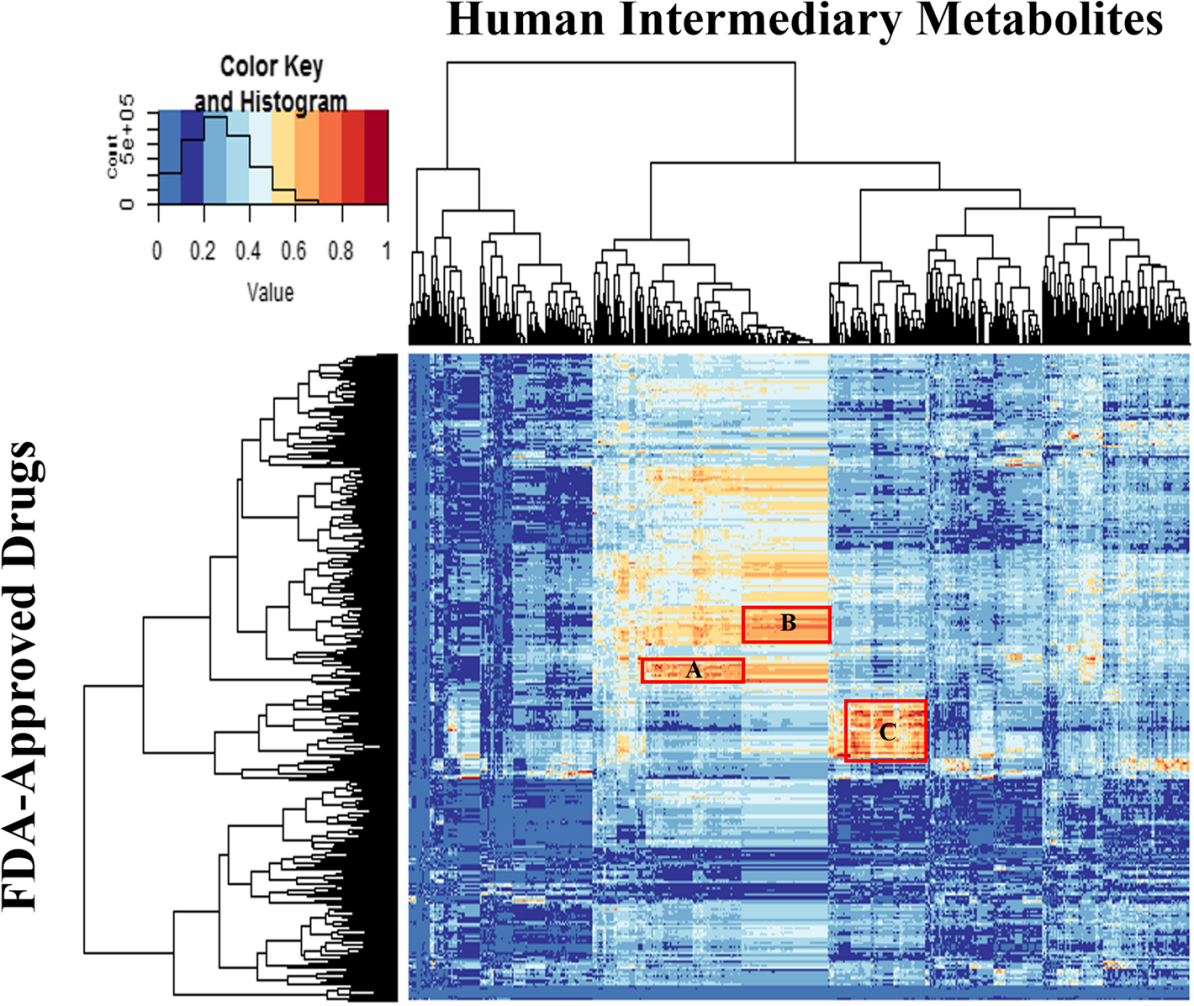
Heat map of 2D structural similarities between the FDA-approved drugs and human intermediary metabolites. The Tanimoto similarity matrix between the 1,861 drugs and 1,110 metabolites encoded by the MACCS key fingerprints. *Red boxes* with the A, B, and C labels indicate the highlighted clusters: > 50 drugs, > 100 metabolites, T_c_ **≥** 0.7 (up to 30%)

We investigated the possibility of using the metabolite-likeness concept for predicting new candidates for enzyme modulators from FDA-approved drugs. To see the global patterns of the metabolite-likeness of a drug space, we first generated a structural similarity matrix between FDA-approved drugs and human intermediary metabolites (Fig. 1). As shown in Fig. 1, we found three interesting clusters (A-C) which show a high overall Tanimoto similarity in the cluster. The metabolites set in cluster A represented purine and pyrimidine containing derivatives, cluster B represented CoA derivatives, and C represented sterols and steroids. The results of cluster B and C are concordant with a previous study [19]. However, the result of cluster A has not been explored. As a result of further investigation, we recognized that almost 30% of drugs in cluster A are antimetabolite class drugs. An antimetabolite is a class of drug that contains structurally similar substances to naturally occurring molecules (i.e., metabolites). Therefore, they interfere with physiological reactions involving their similar metabolites [43]. By definition of an antimetabolite, we decided to use antimetabolite class drugs as a gold standard positive (GSP) set for enzyme modulator prediction.

### Evaluation of the metabolite-likeness on antimetabolite class drug set

To collect a complete set of the antimetabolites, we manually curated a list of antimetabolites from DrugBank [27]. Because an antimetabolite can be mapped to multiple metabolites, we chose a substrate with the highest similarity to the drug from the substrates set of each enzyme only. Moreover, if the antimetabolites have a low similarity to the substrates of their corresponding target enzyme, they might have different mechanisms of actions which are different from the actions of endogenous metabolites. Therefore, we chose the GSP set only if the similarity value was over 0.5. As a result of the GSP selection procedures, the final GSP list consists of 17 antimetabolite drugs, 11 target enzymes, and 15 substrates, exclusively (Table 1).

**Table 1:** Similarity between Antimetabolites and their corresponding human metabolites

Target Enzyme	Substrate	Antimetabolite	Similarity	*P-*value
TYMS	dUMP	Trifluridine	0.82	1.97E-03
		Floxuridine	0.79	6.38E-12
		Gemcitabine	0.69	2.26E-02
		Capecitabine	0.66	9.54E-03
	5,10-Methylene- tetrahydrofolate	Pemetrexed	0.75	3.85E-04
		Raltitrexed	0.75	8.57E-10
		Pralatrexate	0.69	1.40E-09
POLA1,POLB	dATP	Cladribine	0.77	1.76E-08
		Clofarabine	0.72	6.50E-07
		Fludarabine	0.71	1.94E-06
	dGTP	Nelarabine	0.76	3.38E-09
	dCTP	Cytarabine	0.75	7.13E-52
DHFR	7,8-Dihydrofolate	Pemetrexed	0.86	7.40E-05
	(6S)-5,6,7,8-tetra- hydrofolate(2-)	Pralatrexate	0.82	6.48E-22
		Methotrexate	0.78	1.88E-05
RRM1	ADP	Fludarabine	0.8	2.28E-08
		Clofarabine	0.73	5.95E-05
		Cladribine	0.71	1.59E-02
	CDP	Gemcitabine	0.77	6.06E-03
DNMT1	Cytidine	Azacitidine	0.97	6.01E-17
		Decitabine	0.88	8.06E-12
IMPDH1/2	IMP	Ribavirin	0.69	2.66E-05
ENPP1	Deamino-NAD+	Ribavirin	0.69	1.02E-06
ATIC	10-Formyl- tetrahydrofolate	Pemetrexed	0.79	7.73E-06
GART		Pemetrexed	0.79	7.73E-06
NME1/2	dCDP	Gemcitabine	0.76	1.37E-10
XDH	Hypoxanthine	Allopurinol	0.69	7.90E-12

To see if the metabolite-likeness can predict the antimetabolite-target enzyme relation well, we established a subset similarity matrix that contains 15 antimetabolite related substrates (i.e., metabolites) and 1,861 approved drugs using the finalized GSP relationships. Then, we plotted the z-distributions of the similarity scores between each substrate metabolites and the total drugs. As shown in Table 1, all the antimetabolite-substrate similarity relations have a *p-*value lower than 0.05 in the corresponding z-distribution of the substrate metabolites. (Additional file 1: Figure S1). This result indicates that the metabolite-likeness could predict all the GSP relationships with statistically significant similarity values.

### Performance comparison to other DTI prediction methods

To assess the performance of metabolite-likeness for DTI prediction, we compared the performance of metabolite-likeness to known DTI prediction methods: SwissTargetPrediction (STP) [20], TargetNet (TN) [21], and Libdock algorithm of molecular docking [22]. The performance of each method was assessed based on the ROC curve for the GSP relationships (i.e., antimetabolites-target enzymes).

**Fig. 2:**
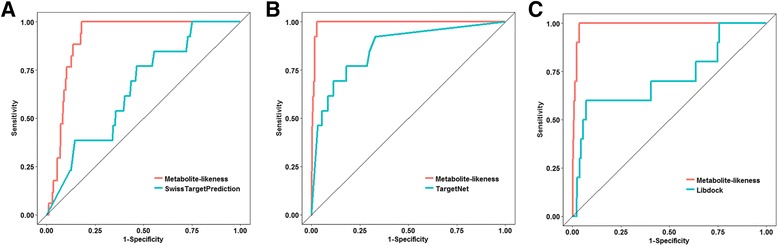
Performance comparison between metabolite-likeness and the other target prediction methods. The receiver operating characteristic (ROC) curves of metabolite-likeness and the other three different methods, (**a**) SwissTargetPrediction, (**b**) TargetNet, and (**c**) Libdock, for the gold standard positive set (antimetabolite-target enzyme)

First, in order to compare the performance between metabolite-likeness and STP fairly, we applied the metabolite-likeness to the DTI prediction in the same way, because we could only get 15 possible targets for each drug from the STP. Comparing the results of the two DTI prediction methods, we obtained 17 GSP relationships from the metabolite-likeness and only 13 GSP relationships from the STP prediction. This result indicates that the metabolite-likeness provided more antimetabolite-target enzyme relations than that of the STP when metabolite-likeness is applied in the same manner as the STP prediction. Figure 2(a) shows the ROC curves calculated with the metabolite-likeness and STP for the GSP relationships. As shown in Fig. 2(a), the area under the ROC curve (AUC) values of the metabolite-likeness and the STP were 0.914 and 0.658, respectively.

Then, we compared the performance between the metabolite-likeness and TN. To this end, we gathered all the probability scores of the predicted targets for all 1,861 of FDA approved drugs. Unlike the STP method, we can obtain the predicted scores for all drugs we used for each target. In the TN method, however, DTI relationships can be predicted only for 620 human proteins. The 4 enzymes of the GSP target, TYMS, DHFR, IMPDH2, and DNMT1, were only overlapped with the 620 human proteins available in the TN. Therefore, we also applied the metabolite-likeness to only the 4 enzymes of the GSP targets for a fair comparison. Figure 2(b) shows that the ROC curves calculated by the metabolite-likeness and TN for the GSP relationships. As shown in Fig. 2(b), the AUC values of the metabolite-likeness and TN were 0.991 and 0.862, respectively.

Lastly, we compared the performance between the metabolite-likeness and molecular docking simulation. Because the structures of some of the target enzymes have not been reported or only parts of the structures were given in a DNA polymerase form, we could not get all of the structures of the target enzymes for the analysis. Thus, among the 11 target enzymes, we only performed the molecular docking simulations, especially using the Libdock algorithm, with DHFR and TYMS to compare the results to our method. The 1,861 FDA-approved drugs were docked into the active sites of DHFR and TYMS. For a fair comparison with the docking results, the metabolite-likeness was applied only to TYMS and DHFR, and the performance was evaluated. Figure 2(c) shows the ROC curves calculated by the metabolite-likeness and molecular docking for the GSP relationships. As shown in Fig. 2(c), the AUC values of the metabolite-likeness and molecular docking simulation were 0.989 and 0.721, respectively. Based on the results of the performance comparison with the other DTI prediction methods, metabolite-likeness showed better performance than all the other methods for the GSP relationships.

### Prediction of drug repositioning candidates for antimetabolite class drugs

**Fig. 3:**
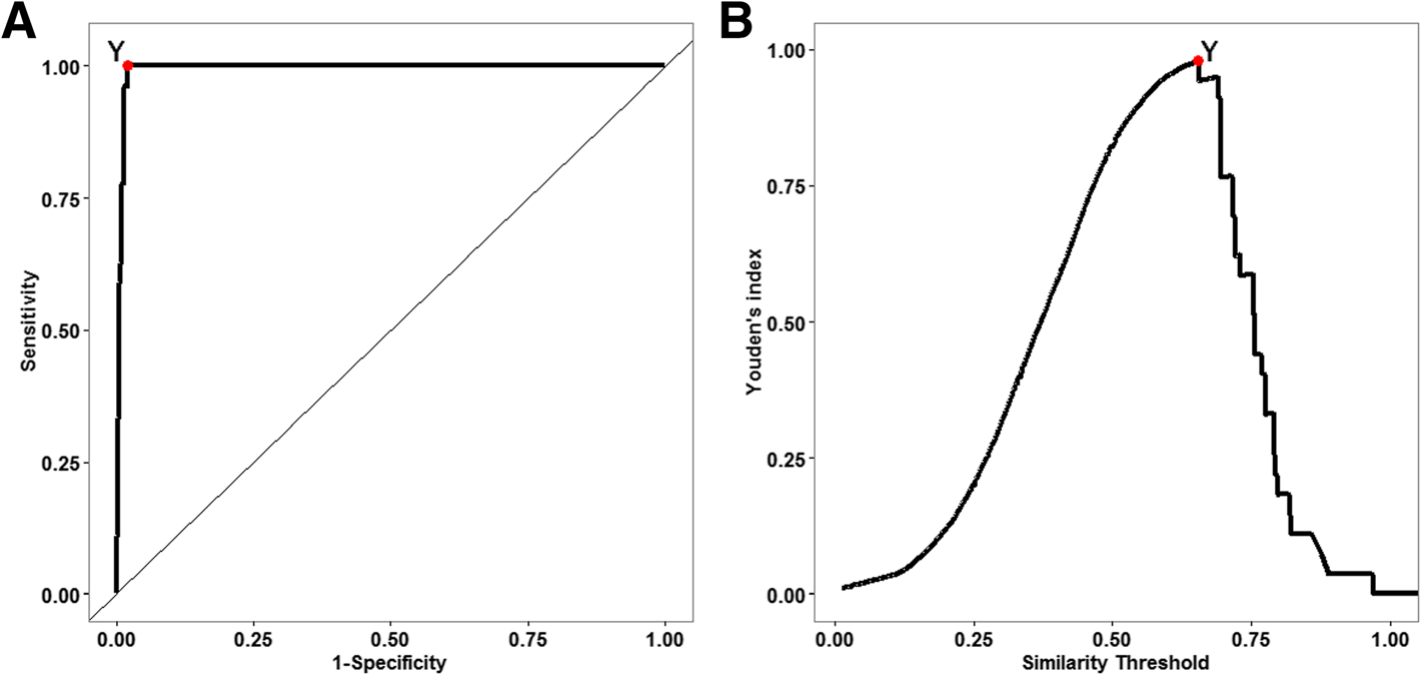
Determination of the optimal similarity threshold for enzyme modulator predictions. The (**a**) ROC curve and (**b**) Profiles of the Youden’s index according to the similarity threshold. *Red points* with the *Y label* indicates the maximum Youden’s index

To determine the optimal similarity threshold for enzyme modulator predictions, we plotted a ROC curve of the metabolite-likeness for all the antimetabolite-target enzyme relationships. As seen in Fig. 3(a), the AUC value is 0.993. Then, the Youden’s index was calculated based on the ROC curve. Figure 3(b) shows that the maximum Youden’s index is 0.979 at a similarity threshold of 0.654. This threshold showed significant classification with a high true positive rate of 1 and a low false positive rate of 0.021. Using this similarity threshold, we obtained new enzyme modulator candidates for the 11 target enzymes of the antimetabolites. Anywhere from 27 to 108 new drug candidates were predicted for each target enzyme of the antimetabolites. In the case of XDH, there was no predicted candidate because only the GSP relation was predicted with the similarity threshold. As shown in Table 2, we summarized only one promising drug candidate as a corresponding enzyme modulator in terms of the highest similarity except for the endogenous ligand and original antimetabolite.

**Table 2:** Selected drug repositioning candidates

Target Enzyme (Disease)^a^	Substrate	Candidate drug (Indicated Disease)	Similarity	Reference
TYMS (Cancer)	5,10-Methylene- tetrahydrofolate	(Levo)leucovorin (Osteosarcoma)	0.889	[44]
POLA1,POLB (Leukemia)	dCTP	Decitabine (Myelodysplastic Syndromes)	0.806	[45, 46]
DHFR (Cancer)	(6S)-5,6,7,8-tetra- hydrofolate(2-)	(Levo)leucovorin (Osteosarcoma)	0.923	[44]
RRM1 (Leukemia)	CDP	Cytarabine (Leukemia)	0.841	[48]
DNMT1 (Leukemia)	Cytidine	Gemcitabine (Cancer)	0.875	[49]
IMPDH1/2 (Chronic Hepatitis C)	IMP	Nelarabine (Leukemia)	0.770	[50]
ENPP1 (Chronic Hepatitis C)	Deamino-NAD+	Vidarabine (Herpes virus infection)	0.781	[51]
ATIC (Mesothlioma)	10-Formyl- tetrahydrofolate	(Levo)leucovorin (Osteosarcoma)	0.969	[44]
GART (Mesothlioma)	10-Formyl- tetrahydrofolate	(Levo)leucovorin (Osteosarcoma)	0.969	[44]
NME1/2 (Cancer)	dCDP	Decitabine (Myelodysplastic Syndromes)	0.806	[47]
XDH (Hyperuricemia)	-	-	-	-

^a^Disease names are designated from the antimetabolite drug’s indicated disease

To support our prediction results, we investigated the relationships between the proposed targets and the corresponding drugs with a literature survey. Among the 10 predicted enzyme-drug relations, 7 (70%) are directly supported by literature evidence. We also found that inhibitors of the predicted target enzymes are a similar class of drug as our prediction. Leucovorin is mainly used for chemotherapy of osteosarcoma. It is not a cytotoxic drug itself but when used with 5-FU, it enhances cancer cell sensitivity to 5-FU. A recent study [44] showed that knockdown of the predicted targets such as TYMS, DHFR, and GART resulted in decreased cytotoxicity of the drug combination in the cancer cell. The relationship between ATIC and Leucovorin is not explicitly described in the literature; however, they might be relevant because one of its inhibitor, methotrexate, co-targets TYMS, DHFR, and GART. Decitabine is known to act on DNA polymerase I (POLA1) [45, 46], and recently, the relationship between the drug and one of its predicted targets, NME 1/2, was reported [47]. Cytarabine is known as a ribonucleotide reductase inhibitor which is a gene product of the predicted target RRM1 [48]. A recent study [49] also showed that Gemcitabine, one of the predicted drugs for DNA methyltransferase 1, does inhibit DNMT1 in HEK293T cells. There are no reports on the effect of Nelarabine for the inhibition of IMPDH1/2. However, because the one class of known IMPDH1/2 inhibitors all resemble its innate metabolite [50], Nelarabine could be another inhibitor of IMPDH1/2. The relationship between ENPP1 and Vidarabine is also unreported; however, it may possible because most investigated NPP inhibitors are adenosine analogs and their derivatives [51]. All these evidences support that metabolite-likeness can predict new drug candidates for the target enzymes of antimetabolites.

### Prediction of drug repositioning candidates for Gaucher disease

To investigate whether metabolite-likeness is applicable to other enzyme groups than just antimetabolites, we applied metabolite-likeness to all drug candidates. Among the drug list within the similarity threshold, we were focused on enzymatic disease-related drugs because an enzyme in an enzymatic disease has a direct disease association. Considering both the metabolite-likeness similarity and enzymatic disease associations, we were able to find miglustat used in Gaucher disease and decided to investigate further. Gaucher disease is a rare autosomal recessive genetic disorder, which is classified as a lysosomal storage disorder [52]. The disease is caused by the accumulation of glucosylceramide due to a deficiency in glucocerebrosidase. Currently, only two drugs, miglustat, and eliglustat have been approved for Substrate Reduction Therapy [53] of Gaucher disease.

**Fig. 4:**
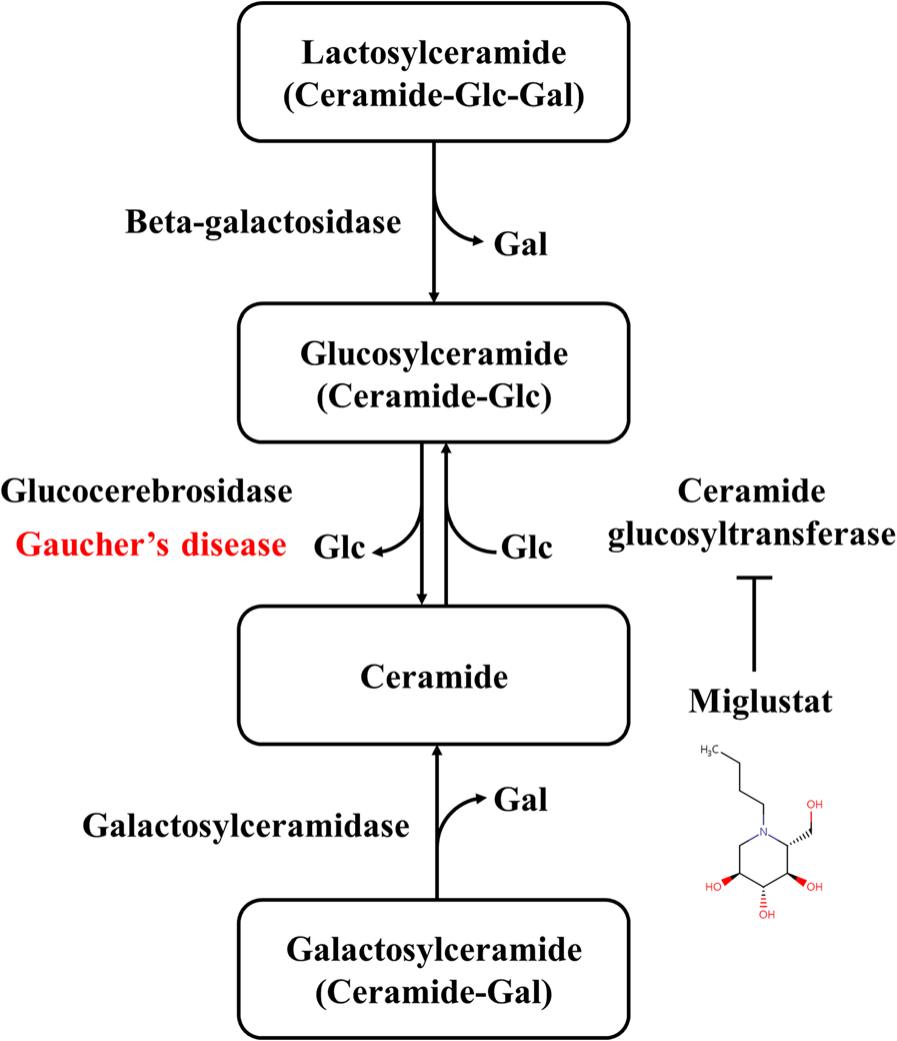
Sphingolipid metabolism pathway in Gaucher disease. Gaucher disease is caused by a deficiency in the glucocerebrosidase enzyme. Miglustat inhibits the ceramide glucosyltransferase enzyme to reduce the glucocerebroside which accumulates in Gaucher disease. Glc, glucose; Gal, galactose

First, we hypothesized that we could repurpose effective drugs with metabolite-likeness that can reduce the substrate by modulating enzymes nearby glucosylceramide. As shown in Fig. 4, only 3 metabolites were identified as similar metabolites with the known drug miglustat. The three metabolites are all located near ceramide (In Fig. 4, Galactosylceramide, Glucosylceramide, and Lactosylceramide). This result implies that miglustat reduces glucosylceramide by modulating glucocerebrosidase.

Next, we examined the new drug candidates list within our threshold. A total of 36 drugs were on the list excluding miglustat. We looked up the indications of all 36 drugs and found that 50% of the drugs (18) were antibiotics and other 50% of the drugs were used for antihypertensive, immunosuppressant, and anti-diabetic indications. These seem like intriguing results supported by the literature. About half of the antibiotics we found were related to aminoglycoside which is also known as aminocyclitol antibiotics (Table 3). In a recent report, aminocyclitol derivatives were reported as efficacious in Gaucher disease [54], and therefore, these antibiotics might be efficacious in Gaucher disease because miglustat, also known as N-butyl-deoxynojirimycin, was first discovered from the nojirimycin class of antibiotics [55].

**Table 3:** Predicted drug repositioning candidates for Gaucher disease

Drug Name	Original Indication	References
Framycetin	Aminoglycosides (Aminocyclitols)	[54]
Amikacin	Aminoglycosides (Aminocyclitols)	
Tobramycin	Aminoglycosides (Aminocyclitols)	
Gentamicin	Aminoglycosides (Aminocyclitols)	
Netilmicin	Aminoglycosides (Aminocyclitols)	
Neomycin	Aminoglycosides (Aminocyclitols)	
Kanamycin	Aminoglycosides (Aminocyclitols)	
Ribostamycin	Aminoglycosides (Aminocyclitols)	
Arbekacin	Aminoglycosides (Aminocyclitols)	
Acarbose	Alpha-glucosidase inhibitors	[56]
Miglitol	Alpha-glucosidase inhibitors	
Bimatoprost	Anti-hypertension	[57–59]
Aliskiren	Anti-hypertension	
Tacrolimus	Immunosuppressant	
Sirolimus	Immunosuppressant	
Everolimus	Immunosuppressant	

Another interesting class of drug in our list was alpha-glucosidase inhibitors (Table 3). The association between alpha-glucosidase and Gaucher disease is not evident; however, we found that this class of drug could be a chemical chaperone for misfolded alpha-glucosidase according to the recent report [56]. Moreover, because a recent repositioning study showed that anti-hypertensive and immunosuppressant class drugs might be efficacious in Gaucher disease [57–59] as well, we expect that the non-antibiotic drugs on our list may be effective in the disease (Table 3).

This evidence supported that our finding is not a fictitious result that metabolite-likeness could be applied to investigate and prioritize drugs that can act similar to innate human metabolites.

## Conclusions

In this study, we addressed the potential of metabolite-likeness for drug repositioning to enzyme related diseases. The novel point of this paper is that new drug target interactions can be predicted with the metabolite-enzyme relationships which could be obtained from metabolic reactions even though there is no drug or chemical interaction information for a particular target. Although several structure-based target prediction methods such as STP [20], TN [21] and the Libdock algorithm of molecular docking [22] are more comprehensive approaches than our method, they do not consider the metabolite-enzyme relationships that could be obtained by the metabolite reactions. Therefore, although metabolite-likeness is a simple method using a similarity measure with metabolite, it has shown better performance than the other methods for an antimetabolite set, which is a drug class with high similarity to a metabolite. To the best of our knowledge, there are no publications that have applied the metabolite-likeness concept to examine possible drug candidates which have a similar mechanism of action as innate metabolites. Furthermore, we believe that we can predict better drug-target interactions if we combine the proposed metabolite-likeness method with the existing comprehensive DTI prediction tool.

Although we explored the metabolite-likeness concept in the existing drug space only, this analysis can be extended to other enzyme associated disease spaces and other chemical spaces. Moreover, the new drug-enzyme interaction prediction method through metabolite-likeness may have more possibilities in predicting drugs that have a good ADMET property because it can predict more metabolite-like drugs. In another aspect of metabolite-likeness, the drug target space could be expanded by a similarity search to innate metabolites of unexplored enzymes. In addition, by applying this analysis on a larger scale, we expect that we could identify potential enzyme modulators in a systematic way. This work would provide new insight into metabolite-likeness for drug-target prediction and drug repositioning.

## Additional file

Additional file 1: Figure S1. Statistical evaluations of metabolite-likeness similarities of the gold standard positive relationships for the corresponding distributions of metabolites (DOCX 125 kb)

## Abbreviations

ADMET: Absorption, Distribution, Metabolism, Excretion, and Toxicity
FDA: The Food and Drug Administration
MACCS: Molecular ACCess System
