# Supplementary material for: Drug repositioning for enzyme modulator based on human metabolite-likeness
Source: BMC Bioinformatics. 2017 May 31;18(Suppl 7):226. doi: 10.1186/s12859-017-1637-5 (PMC5471945; doi:10.1186/s12859-017-1637-5)
Supplement: Additional file 1: Figure S1. — Statistical evaluations of metabolite-likeness similarities of the gold standard positive relationships for the corresponding distributions of metabolites (DOCX 125 kb) [file 12859_2017_1637_MOESM1_ESM.docx]

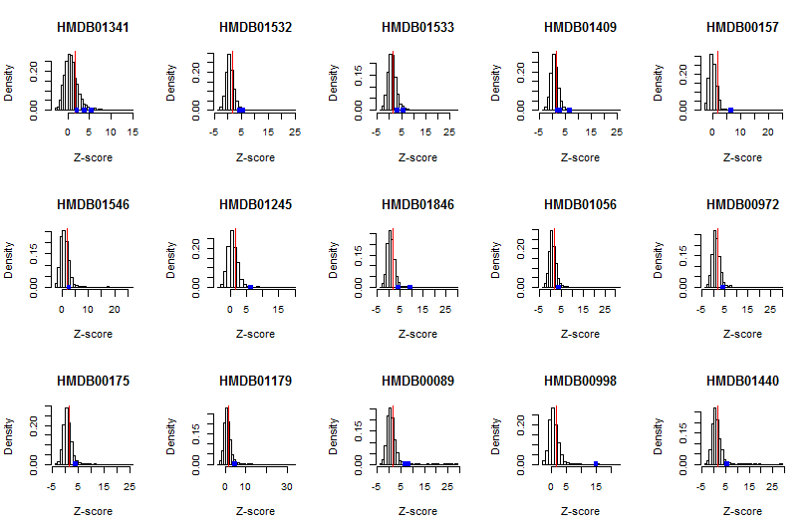


**Additional file 1: Figure S1. Statistical evaluations of metabolite-likeness similarities of gold standard positive relationships in the corresponding metabolite’s distributions**. Statistical evaluations of 26 antimetabolite drug-target enzyme-substrate relations in the z-distributions of the similarity scores between each substrate metabolites and the total drugs. Blue points indicates gold standard relations. Red vertical lines indicates critical z-statistics for right-tailed p-value of 0.05. HMDB IDs indicate the following metabolites: HMDB01341, ADP; HMDB01532, dATP; HMDB01533, 5,10-Methylenetetrahydrofolate; HMDB01409, dUMP; HMDB00157, Hypoxanthine; HMDB01546, CDP; HMDB1245, dCDP; HMDB01846, (6S)-5,6,7,8-tetrahydrofolate(2-); HMDB01056, 7,8-Dihydrofolate; HMDB00972, 10-Formyl-tetrahydrofolate; HMDB00175, IMP; HMDB01179, Deamino-NAD+; HMDB00089, Cytidine; HMDB00998, dCTP; HMDB01440, dGTP.
